# Molecular Detection of *Gurltia paralysans* by Semi-Nested PCR in Cerebrospinal Fluid and Serum Samples from Domestic Cats (*Felis catus*)

**DOI:** 10.3390/ani10071169

**Published:** 2020-07-09

**Authors:** Freddy López-Contreras, Lisbeth Rojas-Barón, Marcelo Gómez, Francisco Morera, Paulina Sepúlveda, Manuel Moroni, Pamela Muñoz, Gerardo Acosta-Jammett, Marcelo Mieres, Jörg Hirzmann, Carlos Hermosilla, Anja Taubert

**Affiliations:** 1 Institute of Pharmacology and Morphophysiology, Austral University of Chile, Valdivia 5090000, Chile; freddy.lopez@postgrado.uach.cl (F.L.-C.); liscrb@gmail.com (L.R.-B.); fjmorera@uach.cl (F.M.); paulina.sepulveda.garcia@gmail.com (P.S.); 2 Institute of Animal Pathology, Austral University of Chile, Valdivia 5090000, Chile; manuelmoroni@uach.cl (M.M.); pamela.munoz@uach.cl (P.M.); 3 Institute of Veterinary Preventive Medicine, Austral University of Chile, Valdivia 5090000, Chile; gerardo.acosta@uach.cl; 4 Institute of Veterinarian Clinic Sciences, Austral University of Chile, Valdivia 5090000, Chile; mmieres@uach.cl; 5 Institute of Parasitology, Justus Liebig University Giessen, 35392 Giessen, Germany; joerg.hirzmann@vetmed.uni-giessen.de (J.H.); carlos.r.hermosilla@vetmed.uni-giessen.de (C.H.); anja.taubert@vetmed.uni-giessen.de (A.T.)

**Keywords:** *Gurltia paralysans*, cerebrospinal fluid, serum, semi-nested PCR, molecular detection

## Abstract

**Simple Summary:**

Feline gurltiosis is a parasitic myelopathy caused by *Gurltia paralysans*. This nematode infects domestic cats without distinction of sex, breed or age, invading the venous system of the spinal leptomeninges and causing vascular congestion that mainly produces paralysis of the pelvic limbs, among other clinical signs of chronic myelopathy. To date, the definitive diagnosis of feline gurltiosis is only possible through post-mortem analysis that shows the location of the parasite in the vasculature of the spinal cord. For this reason, this investigation aimed to detect *G. paralysans* DNA, via semi-nested PCR, in samples of cerebrospinal fluid and serum from 12 domestic cats from potentially endemic areas in southern Chile, with compatible signs of feline gurltiosis. The presence of *G. paralysans*-specific DNA was detected in the cerebrospinal fluid of four out of nine cats and the sera of seven out of seven cats. These results allow us to suggest the implementation of a semi-nested PCR technique as a routine diagnostic test for early and timely detection of feline gurltiosis.

**Abstract:**

*Gurltia paralysans* is an angio-neurotropic metastrongyloid nematode that infects domestic and wild cats, invading the veins of the subarachnoid space of the spinal cord and mainly causing progressive paralysis of the pelvic limbs. The definitive diagnosis of feline gurltiosis can only be achieved by post-mortem examination that reveals the presence of the nematode in the spinal cord vein vasculature. An early diagnosis with conclusive results is required since laboratory and imaging findings are not sufficient. Therefore, the purpose of this study was to detect the presence of *G. paralysans*, via semi-nested PCR, in samples of cerebrospinal fluid (CSF) and the sera of domestic cats naturally infected with the parasite. A total of 12 cats with a diagnosis suggestive of feline gurltiosis were selected, and they underwent a complete neurological and imaging examination. DNA samples were analysed by semi-nested PCR, with universal (AaGp28Sa1/AaGp28Ss1) and specific (Gp28Sa3/Aa28Ss2) primers, for *G. paralysans* (*G. paralysans* 18S rRNA gene, partial sequence; ITS 1, 5.8S rRNA gene, and ITS 2, complete sequence; and 28S rRNA gene, partial sequence) and *Aelurostrongylus abstrusus*, obtaining amplifications of 356 and 300 bp, which indicated the presence or absence of nematode DNA, respectively. The presence of *G. paralysans* was detected in the CSF of four out of nine cats, and the sera of seven out of seven cats. In the sera analysis of five out of seven cats, a mixed infection with *A. abstrusus* was found, despite no alterations of the respiratory tract being observed during the necropsies. It is proposed that serum samples could be more effective than CSF in detecting the parasite by PCR analysis. Sequencing analysis showed high percentages of identity with *G. paralysans*, which indicated the feasibility of detection and the sensitivity/specificity of the method used, suggesting the implementation of semi-nested PCR as a routine diagnostic test for early and timely detection of feline gurltiosis.

## 1. Introduction

**Figure 1:**
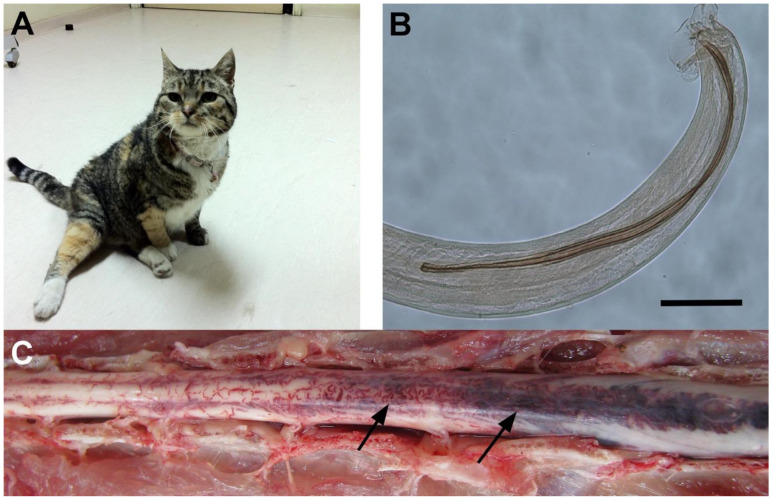
Clinical and pathological findings in a domestic cat with *Gurltia paralysis* infection. Adult domestic cat infected with *G. paralysans* with chronic paraparesis (**A**). Caudal end from an adult male specimen of *G. paralysans* (scale bar: 100 μm) (**B**). Photograph from the lumbar spinal cord segments from a naturally infected cat with *G. paralysans* showing marked submeningeal vascular congestion (arrows) (**C**).

*Gurltia paralysans* is an emerging metastrongiloid nematode (class Nematoda, Order Strongylidae, Superfamily Metastrongyloidea, Family Protostrongylidae, Subfamily Angiostrongylinae), in from domestic cats (*Felis catus*) and wild felids (*Leopardus triginus* and *Leopardus wieddi)*. It has an angio- and neurotropic character that mainly inhabits the veins of the subarachnoid space of the spinal cord [1] (Figure 1A–C). The life cycle of the parasite is unknown, but is probably indirect, involving gastropods as the intermediate host (IH), as in other metastrongyloid nematode species [1,2]. Felines may become infected by ingestion of the IH or a paratenic host (e.g., lizard, bird, rodent) with an infective third-stage larvae. The geographical distribution of *G. paralysans* includes geographic areas of Chile, Argentina, Uruguay, Colombia, Brazil, and recently the island of Tenerife, Spain [2,3,4,5,6,7].

This nematode invades the venous system of the leptomeninges, specifically in the thoracic, lumbar, and sacral spinal cord segments of wild and domestic cats [8,9]. This disposition triggers thrombi, congestion, varicose veins, and consequently severe myelopathies with fatal results, and therefore it has been associated with hind limb paralysis in domestic cats [10,11] (Figure 1C). Generally, the clinical manifestation of feline gurltiosis includes chronic symmetric or asymmetric ataxia of the pelvic limb, ambulatory paraparesis, proprioceptive deficits and atrophy of the pelvic limbs, weight loss, coprostasis, urinary and faecal incontinence, and even death [4]. Reported haematologic findings include mild degrees of anaemia, eosinophilia and thrombocytopenia [12].

Imaging studies through conventional myelography indicate the thinning and obstruction of the dorsal and ventral columns of contrast medium in the thoracolumbar region, as well as diffuse enlargement of the spinal cord in the thoracic, lumbar and sacral regions, observed by computed tomography myelography (Myelo-CT) and MRI [12].

Currently, the presumptive diagnosis of *G. paralysans* infection is based on the medical history of domestic cats with progressive clinical features from potentially endemic areas [3,4,5,6,7,8,9]. Consequently, the definitive intra vitam diagnosis of feline gurltiosis can only be achieved by a post-mortem examination that reveals typical spinal cord lesions and the presence of parasites in the vasculature of the spinal cord (Figure 1C). There is no early diagnosis that would permit suggestions for the administration of timely and reversible treatment of this neglected parasitosis. In addition, no other methods are available for a precise ante-mortem detection of this nematode, and therefore, the objective of this study was to detect the presence of *G. paralysans*, with the implementation of semi-nested PCR as a sensitive and specific molecular diagnostic tool. Additionally, we aimed to evaluate cerebrospinal fluid and sera as appropriate samples for the molecular detection of this nematode.

## 2. Materials and Methods

### 2.1. Selection of Patients

Cerebrospinal fluid (CSF) and serum samples were collected from 12 domestic cats with suggestive diagnoses of feline gurltiosis, presented at the Veterinary Clinical Hospital of the Universidad Austral de Chile (UACh), Valdivia, Chile. All the domestic cats came from rural or peri-urban areas of southern Chile, from habitats considered potentially endemic for feline gurltiosis as reported elsewhere [12]. The selected domestic cats had no previous history of anthelminthic treatments, and presented clinical features coinciding with feline gurltiosis, which included chronic paraparesis/paraplegia, ambulatory/non-ambulatory ataxia, hind limb musculoskeletal atrophy (MPs), decreased muscle tone in the MPs, alteration of spinal reflexes in the MPs, proprioceptive deficits of MPs, urinary incontinence and/or tail atony [9,10,12]. All individuals showing neurological clinical signs that did not correspond to feline gurltiosis and/or with other pathologies, such as spinal trauma, neoplasms, infections or degenerative or metabolic processes, were excluded. No distinction was made regarding breed, sex, age or weight.

### 2.2. Neurological Examination and Sampling

All animals were subjected to a complete neurological examination that included evaluation of the mental status, cranial nerves, posture and gait, postural reactions, spinal reflexes, and determination of pain and palpation [13,14]. Subsequently, serum and CSF from all animals were taken. To achieve this, sedation of each individual cat was performed using a combination of xylazine–ketamine at concentrations of 1 mg/kg and 10 mg/kg intramuscularly (i.m.), respectively (Xylazine 20 mg/mL Xylavet 2% Lab. Alfasan, Worden, Holland/Ketamine Hydrochloride 111.56 mg/mL Ketamil Lab. Ilium, New South Wales, Australia). Blood samples to obtain sera were taken from the external jugular vein (22G × 1½″ needle, Cranberry-Reutter, Santiago, Chile. Cerebrospinal fluid samples were obtained through a puncture of the subarachnoid space at the atlanto-occipital joint level (24G butterfly needle, Channelmed, Santiago, Chile). Samples were collected in sterile Eppendorf tubes without anticoagulants and stored at −80 °C for subsequent molecular analysis.

### 2.3. Imaging Studies

Conventional myelography and Myelo-CT scans were performed on all patients to rule out extradural, intradural/extramedullary and intramedullary pathologies that might be associated with the neurological condition of the patient. In both examinations, the contrast medium was iohexol (657.1 mg/mL Omnipaque™ Lab. Sanofi-Synthelabo, Rio de Janeiro, Brazil) with a dose of 0.5 mg/kg intrathecal. General anaesthesia was applied during both procedures, maintained with ketamine (5 mg/kg) and supplemented with diazepam 0.5 mg/kg).

### 2.4. Necropsy and Extraction of Nematodes

In cases of chronic paraplegia (>3 weeks) without the presence of deep pain, euthanasia was performed with the prior consent of the owners. To do this, the felines were induced with xylazine 2% (1 mg/kg) and ketamine 10% (10 mg/kg) intravenously, then the solution for euthanasia T61^®^ (Intervet International Gmbh, Schwabenheim, Germany) was used. The entire spinal cord was removed from the vertebral canal, and incisions were made in the dura mater to expose the affected blood vessels in the subarachnoid space of the cervical, thoracic, lumbar and sacral regions. Severely affected areas of the spinal cord that contained congestive and/or dilated vessels were dissected. The spinal subarachnoid microvasculature was inspected using a Stemi DV4^®^ (Carl Zeiss Microscopy, Jena, Germany) stereoscopic magnifying glass. Parasites located in these areas were carefully manually extracted using a fine needle, then fixed with 70% ethanol and rinsed in Amman lactophenol (Merck, Darmstadt, Germany) [15]. Finally, they were individually placed on a slide for morphological identification under a light microscope [16].

### 2.5. Extraction and Quantification of Gurltia paralysans DNA

The DNA was extracted from serum and CSF samples using the DNeasy^®^ Blood & Tissue kit (Qiagen, Hilden, Germany), following the manufacturer’s instructions. Purified DNA samples were quantified by Nanodrop^®^ 2000 spectrophotometry (Thermo Fisher Scientific, Waltham, MA, USA) and stored at −20 °C until use.

### 2.6. Molecular Detection of Gurltia paralysans by Semi-Nested PCR

Two PCR tests were carried out, one general and two specifics, for molecular diagnosis of *G. paralysans* and *A. abstrusus*, respectively. First, PCR amplified a common metastrongyloid sequence using universal oligonucleotides U1 (*Aa*Gp28Sa1) and U2 (*Aa*Gp28Ss1). The second specific PCR (semi-nested) differentiated between *G. paralysans* DNA (U2 universal oligonucleotide and *G*. *paralysans*-specific oligonucleotide E2: *Gp*28Sa3) and *A. abstrusus* (U1 universal oligonucleotide and *A. abstrusus*-specific E1: *Aa*28Ss2). These species-specific oligonucleotides were designed using the Bacon Designer Program (Premier Biosoft, Palo Alto, USA) from the multiple alignment of the 28S subunit of the metastrongyloid rDNA [10] (Table 1).

The general PCR mixture was composed of GoTaq Green Master Mix 2X^®^ Polymerase (Promega Corporation, Fitchburg, MA, USA) with a final concentration of 1 × 10 µM of each universal oligonucleotide (U1 and U2), approximately 200 ng of DNA (serum and cerebrospinal fluid) and a sufficient quantity of sterile nuclease-free water to reach a final volume of 25 µL. The same concentrations of components were used for the semi-nested PCRs, taking into account the mixture of oligonucleotides specific for each species (*G. paralysans*: U2/E2; *A. abstrusus*: U1/E1). It must be noted that the semi-nested PCR was performed only on those samples that were positive in the general PCR.

The positive control used in the molecular analyses is represented by a metastrongyloid-derived DNA sample that was cloned into a plasmid vector named K3/K10, prepared at the Institute of Parasitology at the Justus Liebig University Giessen (JLU) in Germany. The negative control was the same reaction mixture with nuclease-free water and no DNA.

Amplification conditions for general PCR consisted of an initial denaturation at 94 °C for 5 min followed by 35 denaturation cycles at 94 °C for 30 s, alignment at 54 °C for 30 s, extension at 72 °C for 30 s, and a final extension at 72 °C for 5 min. Similarly, the amplification conditions for the semi-nested PCRs consisted of initial denaturation at 94 °C for one min, followed by 35 denaturation cycles at 94 °C for 30 s, alignment at 54 °C for 30 s, extension at 72 °C for 30 s, and a final extension at 72 °C for 5 min.

The PCR products were analysed by electrophoresis on 2% agarose gels (Fermelo Biotec, Santiago, Chile) prepared in TBE buffer (Tris-Borate-EDTA), supplemented with intercalating staining agent SYBR^®^ Safe DNA Stain Gel (Invitrogen, Paisley, UK) and revealed in the presence of UV light. Likewise, a 100 bp molecular weight marker (Maestrogen, Hsinchu, Taiwan) was used. The implemented electrophoresis conditions were 90 V and 300 mA for 30 min.

### 2.7. Internal Control Amplification by Real-Time PCR

A segment of the 28S subunit of rDNA specific to the feline gene was amplified [17]. The oligonucleotides used were described as feline 28S rDNA, which amplified a 100 bp fragment (please refer to Table 1).

The PCR mixture consisted of Maxima^®^ SYBR Green/ROX qPCR Master Mix 2X (Thermo Fisher Scientific) at a final concentration of 1 × 300 ng of each oligonucleotide, 100 to 150 ng of feline genomic DNA and a sufficient amount of sterile nuclease-free water for a final volume of 25 µL. Amplification conditions consisted of incubation at 50 °C for 2 min and initial denaturation at 95 °C for 10 min, followed by 40 cycles of denaturation at 95 °C for 15 s and alignment at 60 °C for one min. The cycle was then dissociated at 95 °C for 15 s, 60 °C for 20 s, 95 °C for 20 min, and finally 95 °C for 15 s on a Stratagene Mx3000P^®^ kit (Thermo Fisher Scientific). The maximum difference in fluorescence intensity (ΔRn) was determined using MxPro^®^ software (Version 4.00, Stratagene, Stockport, UK).

### 2.8. Sequencing Analysis

Three CSF amplifications (cats B, F and I) and five sera amplifications (cats A, B, D, E and F) previously positive for *G. paralysans* and *A. abstrusus* were randomly selected to verify their specificity by sequencing. The Silica Bead DNA Gel Extraction^®^ kit (Thermo Fisher Scientific) was used for purification of the amplified products following the manufacturer’s instructions. The results obtained were evaluated through BioEdit version 7.0.9.0 biological sequence editing software and the Basic Local Alignment Search Tool (BLAST) available from the National Center for Biotechnology Information (NCBI).

### 2.9. Statistical Analysis

The McNemar test was used to measure the concordance in *G. paralysans* DNA determination between the samples of CSF and serum with the semi-nested PCR technique. A Kappa coefficient was used to determine the similarity in the determination of *G. paralysans* DNA between samples (K < 0.2 = poor agreement, K ≥ 0.2 to 0.4 = fair agreement, K ≥ 0.41 to 0.6 = moderate agreement, K ≥ 0.61 to 0.8 = good agreement, K ≥ 0.81 to 1.0 = very good agreement). Analyses were performed using the program GraphPad^©^ Software, Inc. (San Diego, CA, USA).

## 3. Results

### 3.1. Biological Samples

All feline patients analysed in this study came from either rural (*n* = 10) or peri-urban (*n* = 2) areas of the Los Ríos and Los Lagos regions, southern Chile, both considered geographically endemic areas of feline gurltiosis [10]. It must be noted that blood samples were taken from all domestic cats, without exception, that met the inclusion/exclusion criteria established for this investigation. However, the CSF sampling could only be carried out in nine of the participants (i.e., cats A → I) because their owners did not authorize the administration of general anaesthesia to the remaining three (cats J, K and L).

### 3.2. Neurological Observations

Neurological evaluation results were similar for all individuals, with neuroanatomical lesions being found in different spinal cord segments, corresponding to a multifocal condition. The main neurological signs observed included chronic paraplegia (>3 weeks), loss of spinal reflexes of the hind limbs, ambulatory and non-ambulatory paraparesis, ataxia, and proprioceptive deficits of the hind limbs. The absence of deep and superficial pain and the presence of paraplegia were identified in seven of the affected domestic cats (cats A, B, D, E, G, H and I), which determined the decision of euthanasia by the patient owners (Table 2).

### 3.3. Imaging Analysis

The images obtained by Myelo-CT showed diffuse enlargement of the spinal cord in the thoracic, lumbar and sacral regions, which included focal deformations of the spinal cord, such as flattening of its dorsolateral region, dorsal or ventral dilation of the subarachnoid space and irregular edges of the spinal cord silhouette. Conversely, post-contrast CT showed congested perimedular veins in the vertebral venous plexus of the caudal lumbar region. All imaging studies showed extensive and diffuse nonspecific lesions of the thoracolumbar and lumbosacral areas, which were correlated with the neurological signs (see Table 2).

### 3.4. Pathological Findings

The necropsies of the seven above-mentioned patients (cats A, B, D, E, G, H and I) revealed macroscopic alterations of the spinal cords coincident with feline gurltiosis, showing abundant inflammation of the meninges and spinal cord at the thoracolumbar and lumbosacral levels, as well as submeningeal venous congestion and significant vascular tortuosity. The presence of adult male and female parasites was observed in the veins of the subarachnoid space of the lumbosacral region of the spinal cord in four cats, identified as A, B, E and H.

### 3.5. Molecular Detection of Feline Gurltiosis

**Figure 2:**
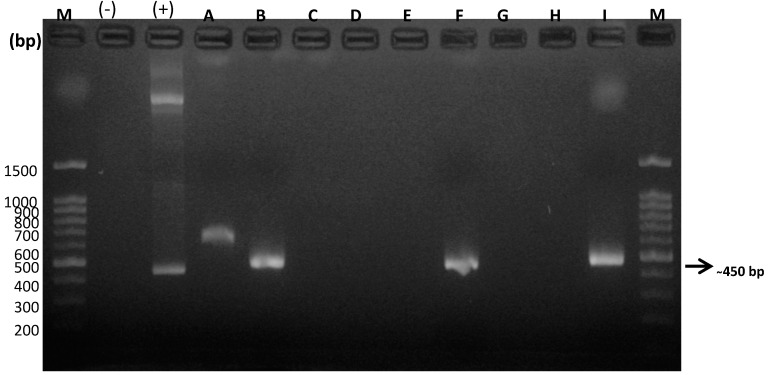
Amplification products obtained by PCR in a general assay for DNA samples obtained from cerebrospinal fluid (CSF) of domestic cats. M: Molecular weight marker; (−): Negative Control; (+): Positive control; bp: base pairs; A → I: domestic cats analysed in this reaction.

**Figure 3:**
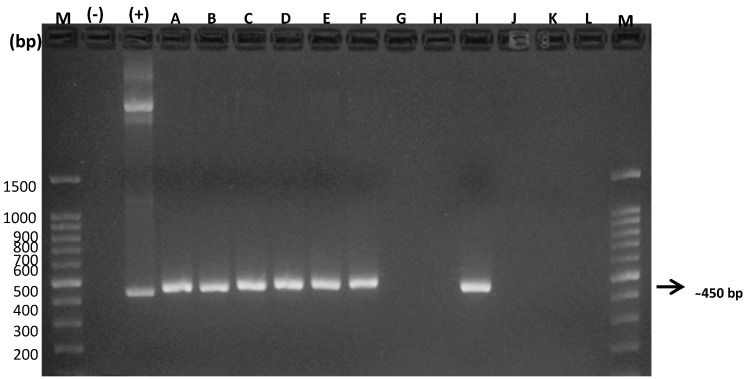
Amplification products obtained by PCR in a general assay for DNA samples obtained from serum of domestic cats. M: Molecular weight marker; (−): Negative control; (+): Positive control; bp: base pairs; A → L: domestic cats analysed in this reaction.

The concentrations of DNA extracted from the biological samples ranged from 11.4 ng/µL to 27.9 ng/µL, with acceptable purity indices, which ensured their viability, and therefore their amplification, allowing them to be considered as completely intact DNA to be used in subsequent PCRs. Consequently, molecular analysis through general PCR amplified a common metastrongyloid sequence of approximately 450 bp. This procedure was applied to the DNA samples purified from the CSF and serum taken from the feline patients considered in this study (Figure 2 and Figure 3).

**Figure 4:**
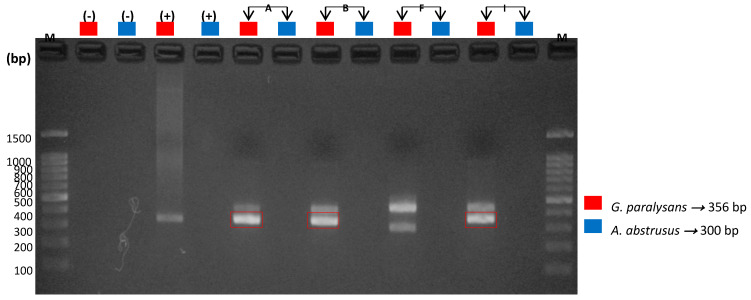
Molecular detection of *G. paralysans* and *A. abstrusus* by semi-nested PCR in samples of cerebrospinal fluid (CSF) from domestic cats. M: Molecular weight marker; (−): Negative control; (+): Positive control; bp: base pairs; A, B, F and I: domestic cats analysed in this reaction.

**Figure 5:**
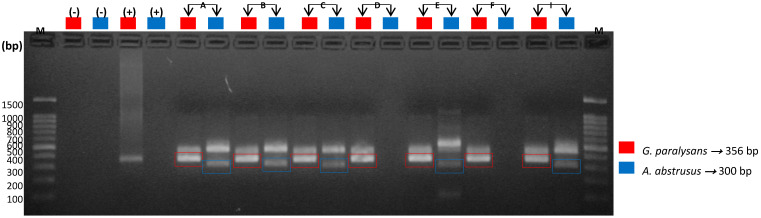
Molecular detection of *G. paralysans* and *A. abstrusus* by semi-nested PCR in serum samples from domestic cats. M: Molecular weight marker; (−): Negative Control; (+): Positive Control; bp: base pairs; A, B, C, D, E, F and I: domestic cats analysed in this reaction.

The following semi-nested specific PCR for each of these species revealed DNA amplification of *G. paralysans* and *A. abstrusus*, which definitively complements and verifies the presence of these nematodes in domestic cats. It must be noted that the species-specific semi-nested PCR analysis was performed only on those DNA samples that tested positive for general PCR, meaning that only four (4/9) of the CSF samples from the domestic felines, identified as A, B, F and I, were evaluated in this system, and the same for the seven (7/12) serum samples from the domestic felines identified as A, B, C, D, E, F and I. *G. paralysans*-specific oligonucleotides *Aa*Gp28Ss1/*Gp*28Sa3 allowed the amplification of a 356 bp fragment, while the *A. abstrusus*-specific oligonucleotides *Aa*28Ss2/*Aa*Gp28Ss2 amplified a 300 bp fragment, as shown in Figure 4 and Figure 5, respectively.

On the other hand, the analysis of the feline 28S rDNA gene using qPCR revealed the specificity of the reaction and amplification, demonstrating that it was indeed feline genomic DNA and not the amplification of a non-specific product, artefact or false positive.

### 3.6. Sequencing Analysis

To verify that the amplification product of the PCR assays belonged mainly to *A. abstrusus* and *G. paralysans*, DNA samples of CSF from patients B, F and I were analysed by sequencing. The serum DNA samples from individuals A, B, D, E and F were analysed in the same way. Verification of these sequences through BLAST (NCBI) showed a high percentage of identity corresponding to *G. paralysans*, which was reflected in the high sensitivity and specificity of the diagnostic method used (Table 3).

### 3.7. Statistical Analysis

To compare the differences in *G. paralysans* DNA determination between the CSF and serum samples, using the semi-nested PCR technique, *X*^2^ = 1.33 was determined as a test statistic, with *p* = 0.25 and df = 1. The McNemar test also indicated there were no significant differences between results of the semi-nested PCR for the CSF and the serum (*p* = 0.24). The Kappa coefficient indicated fair agreement (K = 0.372) between the results of the two samples.

## 4. Discussion

Overall, the findings from the neurological evaluation of affected domestic cats were coincident with those reported by Gómez et al. [10], Rivero et al. [5], Moroni et al. [4], Togni et al. [6] and Mieres et al. [12], suggesting that these clinical manifestations corresponded well with feline gurltiosis. However, Alzate et al. [2] also described similar clinical signs, except for the loss of thoracic and lumbosacral spinal reflexes, in five of six patients evaluated (5/6). It is proposed that the clinical signs of these patients would be mainly associated with the mechanical compression effects caused by subarachnoid venous congestion, since the presence of the nematodes and lesions that are generated can lead to different degrees of spinal compression, as demonstrated previously [4,6]. Similarly, the imaging findings were in line with those reported by Mieres et al. [12], who described the evident presence of multifocal anatomic lesions ranging from the T3 to the Cd5 spinal cord segments. These results were also correlated with the neurological signs findings, and since these characteristics do not denote a specific type of spinal cord pathology and/or spinal column associated with other pathologies, neoplasia, infections and degenerative processes were ruled out, consequently suggesting a prediagnosis of feline gurltiosis in these individuals.

The necropsies carried out showed the presence of adult males and gravid females of *G. paralysans*, with adult forms of both sexes found in cats A, B, E and H (4/7). These findings coincide again with Mieres et al. [12] and Moroni [4], and there are other reported data that describe the finding of adult parasites within the blood vessels of the subarachnoid space of all animals [2,4,6,16,18].

It must be highlighted that, although a presumptive intra vitam diagnosis of *G. paralysans* infection can be made based on a clinical history of paraparesis and progressive chronic paraplegia in domestic cats from potentially highly endemic areas, until now the definitive diagnosis of feline gurltiosis could only be made through a post-mortem examination, which reveals the presence of nematodes in the vasculature of the spinal cord. The laboratory and imaging results do not provide a definitive diagnosis; however, the spinal patterns of inflammation observed by Myelo-CT could be of great importance as well [12], hence the need to explore detection via the use of molecular tools in analysing the CSF and serum DNA from domestic cats naturally infected with this clearly neglected parasite.

As shown in Figure 1, Figure 2, Figure 3, Figure 4 and Figure 5, the DNA extraction and purification method from serum and CSF samples was quite appropriate, since it obtained DNA concentrations that, although relatively low, were sufficient to detect the presence of the parasite without neglecting the inherent high sensitivity and specificity of the PCR technique, making it an ideal detection method for the future. As mentioned, two PCR assays were established, one general and two species-specific, for molecular diagnosis. In the first PCR assay, which served as a template, universal oligonucleotides were used to amplify a common sequence of metastrongyloid nematodes, which allowed for the amplification of a segment of approximately 450 bp. This molecular procedure is based on the amplification of identical conserved sequences of *G. paralysans* and *A. abstrusus*, both considered metastrongyloid nematodes of felid species. In this case, only CSF DNA samples from cats A, B, F and I (Figure 1), and the serum DNA samples from cats A, B, C, D, E, F and I (Figure 2), were positive, which suggests that only these samples may contain *G. paralysans* DNA and, therefore, the typical manifestation of feline gurltiosis, leading to the decision to consider only positive samples for subsequent tests.

The specific oligonucleotides used in the semi-nested PCR analyses were designed from a multiple alignment of the 28S subunit of the metastrongyloid rDNA [10,19]. This assay allowed for differentiation between *G. paralysans* and *A. abstrusus* DNA, taking into account the D2–D3 regions of the 28S rDNA subunit of *G. paralysans* selected for the design of this test, demonstrating at the same time its high specificity and ability to detect and discriminate between these parasites.

As a result of the semi-nested PCR amplification in this study, it was observed that only cats A, B, F and I (4/9), in the case of CSF-derived DNA, were positive for *G. paralysans*, as shown in Figure 3, where the corresponding amplification of 356 bp was evident. Meanwhile, Figure 4 shows the amplification of fragments of 300 and 356 bp in the serum DNA samples of feline patients A, B, C, E and I (5/7), which means that these cats were positive for both parasite species (i.e., *A. abstrusus* and *G. paralysans*), respectively, and therefore a mixed coinfection prevailed, as established by Mieres (2013) [12], despite no observation of alterations in the respiratory tract during the necropsies that might have suggested the presence of the lungworm *A. abstrusus* [17,20,21]. In the case of feline patients D and F (2/7), there was only amplification of a 356 bp fragment, and therefore, it was determined that they were exclusively infected with *G. paralysans*.

With regards to internal control verification, the analysis of the feline 28S rDNA gene (Figure 5) confirmed that the amplification of the biological samples used corresponded to felid-derived DNA, thus excluding amplification of false positives or reaction artefacts.

The analysis of concordance between the molecular results and the autopsy findings described above showed the presence of adult parasites within the subarachnoid blood vessels in cats A, B, E and H (4/7). However, out of these four individuals, *G. paralysans* DNA was only detected in the serum DNA samples originating from cats A, B and E (3/4), and in CSF DNA samples from cats A and B (2/4). In the latter group, the genetic material clearly corresponded to individuals with a higher degree of tissue damage, which could explain the presence of nematode DNA fragments in the submeningeal space, despite *G. paralysans* being an intravascular parasite. It is suspected that in individual H, which had pre-adult larval forms found by necropsy that led to it being definitely diagnosed with feline gurltiosis without successful amplification by PCR, perhaps the DNA concentration was insufficient, or that DNA degradation or the presence of inhibitors carried over from the purification procedure prevented adequate amplification.

In contrast to the present study, other authors have used the 28S rDNA gene to amplify DNA fragments from different metastrongyloid nematodes, such as Mohammad et al. [22], who used first-stage larvae (L1) of *Angiostrongylus vasorum* to amplify the ITS2 region of the 28S gene using conventional PCR. Similarly, Traversa et al. [23] amplified the ITS2 region of the 28S gene in L1 larvae of *A. abstrusus* using conventional PCR, while Jefferies et al. [24] used real-time PCR to amplify the same region but with third-stage larvae (L3) of *A. vasorum*. Other authors used other DNA ribosomal sequence subunits of metastrongyloid nematodes to amplify different partial regions of the 18S gene of *Angiostrongylus cantonensis*, from samples of mucus secreted by slug intermediate hosts, using conventional PCR [25,26]. The amplification of the ITS1 and mtCOI regions of *A. abstrusus* from L3 larvae using nested PCR [27] has been described. Eamsobhana et al. [28] used CSF samples from human patients with eosinophilic meningitis to amplify a native 66 kDa protein from *A. cantonensis*, using conventional PCR. Likewise, Moreira et al. [21] amplified the mtCOI region of *A. cantonensis* in L3, also by using conventional PCR.

Some authors have suggested the possibility of detecting metastrongyloid DNA in other types of biological samples, such as Mohammad et al. [22], who amplified the ITS2 region of rDNA from *A. vasorum* L1 found in faecal samples of wild foxes and domestic dogs from Switzerland, Norway and Denmark, achieving 17 positive results from the same number of samples studied, which were previously identified as positive by coprology tests. Similarly, Traversa et al. [23] amplified the ITS2 between the 5.8S and 28S regions of the rDNA sequence of *A. abstrusus* L1, found in faecal and pharyngeal swabs from cats clinically apparent as aelurostrongylosis and coming from endemic areas of central Italy, obtaining positive results in 24 samples (24/30), previously confirmed by the direct smear method; 28 positive samples (28/30) previously confirmed by the flotation method; and 29 positive samples (29/30) in pharyngeal swabs confirmed by the Baermann method.

It seems noteworthy to mention that in this study, it was not possible to successfully identify *G. paralysans*-derived DNA in faecal samples, since it has not been possible so far to identify its eggs by routine coproparasitological tests. Preliminary data indicating the presence of *G. paralysans* DNA in bronchoalveolar lavage were also recorded, but it is necessary to increase the number of samples and their volume to validate these preliminary results. Concomitantly, Eamsobhana et al. [28] used CSF samples from patients diagnosed with eosinophilic meningitis to amplify a native 66 kDa protein from *A. cantonensis*, obtaining four positive samples (4/10) and developing a specific immunoblot as a “gold standard” test. In contrast, Da Silva et al. [29] amplified *Angiostrongylus costaricensis* genomic DNA from three serum samples of human patients diagnosed with abdominal angiostrongiliasis by histopathology. These samples were positive by PCR, amplifying a fragment of approximately 232 bp. Unlike the serum sample results shown here, Da Silva et al. [29] achieved amplification in all samples; however, the former authors used a lower number (*n* = 3) than that evaluated in this study (*n* = 12).

The results obtained for the molecular detection of *G. paralysans* suggest that serum could be more effective for the detection of species-specific DNA, when compared to CSF samples. *G. paralysans* is known to be an intravascular and non-submeningeal living parasite, even though detection of DNA fragments in these four samples of CSF was also achieved, which suggests the possibility of the release of fragments through the blood–brain barrier due to vascular dilation and increased permeability occurring after tissue damage [30].

Since it is essential to demonstrate the veracity of the molecular diagnosis, the bioinformatic analyses demonstrated that the sequencing of randomly selected samples showed that they do belong to *A. abstrusus* and *G. paralysans*, respectively. Furthermore, the results obtained by BLAST analysis, available in the database of NCBI, revealed a high percentage of identity (between 96.69% and 100%) with *G. paralysans* when compared to sequences in the database. The same occurred in the case of the query sequences of *A. abstrusus*, in which the identity percentages were over 96% similar to previously reported sequences [10].

In this sense, both methods seem to be similar, since no significant differences are found in sequence analysis; however, the differences in the molecular tests regarding the type of biological sample analysed are considered important. All of these data reflect the high sensitivity and specificity of the applied novel molecular diagnostic method, allowing us to suggest its implementation as a useful tool in the routine diagnosis of the neglected condition feline gurltiosis.

Finally, statistical analysis showed that the results were not statistically significant, since the McNemar test were greater than the value of *p* = 0.25. Therefore, the alternative hypothesis was rejected. However, the K value being below 0.4 indicated little agreement between the results of the CSF and serum samples. This indicates that the results for the serum samples are not comparative to those of the CSF samples via semi-nested PCR examination. The incorporation of a larger number of samples is suggested for the obtaining of more conclusive results in the near future.

## 5. Conclusions

It was possible to identify the presence of *G. paralysans*-specific DNA by semi-nested PCR in the serum and CSF samples derived from domestic cats from highly endemic areas naturally infected with this neurotropic parasite. Serum samples were found to be more effective for the molecular diagnosis of feline gurltiosis; however, this result is not statistically significant, and thus incorporation of a larger number of samples for further detailed investigations is recommended. The results for the serum and CSF samples, which were both positive, were in agreement with previously evaluated clinical and pathological findings. The absence of CSF DNA fragments could be associated with the fact that the parasite has an intravascular location in vivo, and there being no release of parasites into the subarachnoid space; nonetheless, *G. paralysans*-derived antigens might reach this neural space, but future studies are needed to clarify this assumption. The use of semi-nested PCR is recommended as an early and timely diagnostic test in the detection of feline gurltiosis.

## Figures and Tables

**Table 1:** Universal and species-specific primer set for the detection of *Gurltia paralysans* and *Aelurostrongylus abstrusus* [10,17].

Name	Sequence (5′→3′)	Target Gene	Amplicon Size
Universal, U1:AaGp28Sa1-R	AGGCATAGTTCACCATCT	Common sequence metastrongyloidea	~450 bp
Universal 2, U2:AaGp28Ss1-F	CGAGTRATATGTATGCCATT		
Specific 1, E1: Aa28Ss2-F	CGTTGATGTTGATGAGTATC	*Aelurostrongylus abstrusus*	300 bp
Specific 2, E2: Gp28Sa3-R	TCTTGCCGCCATTATAGTA	*Gurltia paralysans*	356 bp
rDNA specific for feline gene-Forward	AGCAGGAGGTGTTGGAAGAG	rDNA 28S	100 bp
rDNA specific for feline gene-Reverse	AGGGAGAGAGCCTAATTCAAAGG		

**Table 2:** Clinical, macroscopic and molecular findings in domestic feline patients with a presumptive diagnosis of feline gurltiosis.

ID	Neurolocation	Neurological Examination					Macroscopic Findings	CSF PCR	Serum PCR
		Ataxia	Paraplegia	Superficial Pain	Deep Pain	Caudal/Anal Tone			
**A**	T3–L3, L4–Cd5	YES	YES	NO	NO	NO	Spinal cord inflammation Subarachnoid venous congestion Spinal cord deformation Presence of intravascular parasites	+	+
**B**	T3–L3, L4–Cd5	YES	YES	NO	NO	NO	Spinal cord inflammation Subarachnoid venous congestion Spinal cord deformation Presence of intravascular parasites	+	+
**C**	T3–L3	YES	NO	YES	YES	YES	ND	−	+
**D**	T3–L3, L4–Cd5	YES	YES	NO	NO	NO	Spinal cord inflammation Subarachnoid venous congestion Spinal cord deformation	−	+
**E**	T3–L3, L4–Cd5	YES	YES	NO	NO	NO	Spinal cord inflammation Subarachnoid venous congestion Spinal cord deformation Presence of intravascular parasites	−	+
**F**	T3–L3, L4–Cd5	YES	NO	YES	YES	NO	ND	+	+
**G**	T3–L3, L4–Cd5	YES	YES	NO	NO	NO	Spinal inflammation Subarachnoid venous congestion Spinal cord deformation	−	−
**H**	T3–L3, L4–Cd5	YES	YES	YES	YES	NO	Spinal cord inflammation Subarachnoid venous congestion Spinal cord deformation Presence of intravascular parasites	−	−
**I**	T3–L3, L4–Cd5	YES	YES	NO	NO	NO	Spinal cord inflammation Subarachnoid venous congestion Spinal deformation	+	+
**J**	L4–Cd5	YES	NO	YES	YES	NO	ND	ND	−
**K**	L4–Cd5	YES	NO	YES	YES	NO	ND	ND	−
**L**	L4–Cd5	YES	NO	YES	YES	NO	ND	ND	−

ND: not done; (+): positive result; (−): negative result. T3–L3: thoracic lumbar spinal cord segments. L4–Cd5: lumbosacral-caudal spinal cord segments.

**Table 3:** Results of the sequencing analysis for *G. paralysans* using BLAST search (NCBI).

Sample	Cat	Description	Max. Score	Total Score	Query Coverage	E-Value	% Identity	Accession
CSF	B	*G. paralysans* 18S rRNA gene, partial sequence; ITS 1, 5.8S rRNA gene and ITS 2, complete sequence; and 28S rRNA gene, partial sequence	292	292	99%	2 × 10^−75^	100%	JX975484.2
CSF	F	*G. paralysans* 18S rRNA gene, partial sequence; ITS 1, 5.8S rRNA gene and ITS 2, complete sequence; and 28S rRNA gene, partial sequence	468	468	100%	6 × 10^−128^	100%	JX975484.2
CSF	I	*G. paralysans* 18S rRNA gene, partial sequence; ITS 1, 5.8S rRNA gene and ITS 2, complete sequence; and 28S rRNA gene, partial sequence	333	333	100%	1 × 10^−87^	100%	JX975484.2
Serum	A	*A. abstrusus* 28S rRNA gene	246	246	85%	4 × 10^−61^	96%	AM039759.1
		*G. paralysans* 18S rRNA gene, partial sequence; ITS 1, 5.8S rRNA gene and ITS 2, complete sequence; and 28S rRNA gene, partial sequence	654	654	100%	0	100%	JX975484.2
Serum	B	*G. paralysans* 18S rRNA gene, partial sequence; ITS 1, 5.8S rRNA gene and ITS 2, complete sequence; and 28S rRNA gene, partial sequence	501	501	100%	6 × 10^−138^	100%	JX975484.2
		*A. abstrusus* 28S rRNA gene	564	564	99%	9 × 10^−157^	100%	AM039759.1
Serum	D	*G. paralysans* 18S rRNA gene, partial sequence; ITS 1, 5.8S rRNA gene and ITS 2, complete sequence; and 28S rRNA gene, partial sequence	222	222	100%	2 × 10^−54^	100%	JX975484.2
Serum	E	*G. paralysans* 18S rRNA gene, partial sequence; ITS 1, 5.8S rRNA gene and ITS 2, complete sequence; and 28S rRNA gene, partial sequence	200	200	92%	1 × 10^−47^	96,69%	JX975484.2
		*A. abstrusus* 28S rRNA gene	176	176	89%	1 × 10^−47^	98%	AM039759.1
Serum	F	*G. paralysans* 18S rRNA gene, partial sequence; ITS 1, 5.8S rRNA gene and ITS 2, complete sequence; and 28S rRNA gene, partial sequence	182	182	98%	3 × 10^−42^	100%	JX975484.2
